# Development and validation of early prediction models for new-onset functional impairment in patients after being transferred from the ICU

**DOI:** 10.1038/s41598-024-62447-8

**Published:** 2024-05-24

**Authors:** Zewei Xiao, Limei Zeng, Suiping Chen, Jinhua Wu, Haixing Huang

**Affiliations:** 1 Shantou University Medical College, Shantou, 515000 People’s Republic of China; 2 Department of Nursing, First Affiliated Hospital of Shantou University Medical College, Shantou, 515000 People’s Republic of China

**Keywords:** Intensive care unit, Functional impairment, Activities of daily living, Prediction model, Machine learning, Diagnosis, Disease prevention, Health services, Quality of life, Neurological disorders, Trauma, Risk factors

## Abstract

A significant number of intensive care unit (ICU) survivors experience new-onset functional impairments that impede their activities of daily living (ADL). Currently, no effective assessment tools are available to identify these high-risk patients. This study aims to develop an interpretable machine learning (ML) model for predicting the onset of functional impairment in critically ill patients. Data for this study were sourced from a comprehensive hospital in China, focusing on adult patients admitted to the ICU from August 2022 to August 2023 without prior functional impairments. A least absolute shrinkage and selection operator (LASSO) model was utilized to select predictors for inclusion in the model. Four models, logistic regression, support vector machine (SVM), random forest (RF), and extreme gradient boosting (XGBoost), were constructed and validated. Model performance was assessed using the area under the curve (AUC), accuracy, sensitivity, specificity, positive predictive value (PPV) and negative predictive value (NPV). Additionally, the DALEX package was employed to enhance the interpretability of the final models. The study ultimately included 1,380 patients, with 684 (49.6%) exhibiting new-onset functional impairment on the seventh day after leaving the ICU. Among the four models evaluated, the SVM model demonstrated the best performance, with an AUC of 0.909, accuracy of 0.838, sensitivity of 0.902, specificity of 0.772, PPV of 0.802, and NPV of 0.886. ML models are reliable tools for predicting new-onset functional impairments in critically ill patients. Notably, the SVM model emerged as the most effective, enabling early identification of patients at high risk and facilitating the implementation of timely interventions to improve ADL.

## Introduction

With advances in critical care medicine, the mortality rate among critically ill patients continues to decline, allowing more individuals to be successfully discharged from the intensive care unit (ICU)^1^. However, a subset of patients experiences new-onset functional impairment post-discharge, which impacts their daily living activities^2–4^. Prior research indicates that functional impairment affects 32–59.3% of critically ill patients within 6 months of discharge^5–8^, with 22–40% continuing to experience impairment beyond 6 months^7–10^. Despite potential improvements in functional status over time, the prevalence of functional impairment among critically ill patients warrants attention. Patients who survive the ICU with functional impairments necessitate ongoing medical and nursing care, diminishing their quality of life and increasing the burden on caregivers and society^11–13^. Numerous studies have linked the onset of functional impairment in critically ill patients to various risk factors, including disease severity, age, mechanical ventilation (MV) use, delirium, fractures, and strokes^14–16^. Utilizing these risk factors, four prognostic studies have been undertaken to predict patients at elevated risk for developing functional impairments post-discharge^6,7,17,18^, and thus implementing active early interventions such as neuromuscular electrical stimulation therapies, hormonal therapies, and early mobilization during the ICU stay^14,19^.

Machine learning (ML) offers sophisticated computational and data mining capabilities for identifying associations among variables within complex, high-dimensional datasets. In recent years, ML models have been widely used in medical research and have shown excellent performance^20–22^. Yet, many existing ML models operate as black boxes, with opaque computational processes that limit their broader application^23,24^. The demand for transparent ML models is evident in medical research, particularly given the high stakes involved in patient safety. Recently developed algorithms for interpreting ML models enhance our understanding of their computational processes and bolster user confidence in their application^25,26^.

This study presents the development of an interpretable predictive model for new-onset functional impairment in critically ill patients, utilizing easily available clinical data.

## Methods

### Study design and setting

We conducted a prognostic study to identify the optimal clinical prediction model by comparing the performances of four models: logistic regression, random forest (RF), support vector machine (SVM), and extreme gradient boosting (XGBoost). Data for this study were derived from four ICUs within a Chinese tertiary care hospital. The study encompassed four ICUs: a general ICU, a coronary care unit (CCU), an emergency ICU (EICU), and a neurosurgical ICU (NSICU). We collected patients' demographic and clinical data, including diagnoses, comorbidities, treatments, medications, and laboratory results, from hospital records. Diagnoses were recorded using the 10th Revision of the International Classification of Diseases (ICD-10) codes. This study adhered to the Transparent Reporting of a Multivariable Prediction Model for Individual Prognosis or Diagnosis (TRIPOD) guidelines for reporting prognostic studies^27^. Ethical approval for the study was granted by the review board of the First Affiliated Hospital of Shantou University Medical College (approval number B-2023-092).

### Study population

Patient data from the four ICUs, admitted between 1 August 2022 and 20 August 2023, were retrospectively retrieved from the clinical information system. The target population addressed by the prediction models were adult patients who were independent in activities of daily living (ADL) before hospitalization and who were admitted to the ICU and survived at least 2 days. In the hospitals where this study was conducted, patients were required to receive an ADL assessment and medical history review on admission. We established three inclusion criteria to ensure baseline characteristic homogeneity: (1) age 18 years or older, (2) ICU stay exceeding 48 h, and (3) first-time ICU admission. To predict new-onset functional impairment post-ICU admission, we excluded patients with pre-existing impairments or known risk factors for such impairments: (1) a Barthel Index (BI) ≤ 60 at admission, (2) pre-ICU diagnoses of Parkinson's disease, stroke, epilepsy, or mental disorders, and (3) a history of craniocerebral trauma or surgery.

### Predictors

Potential predictors were selected by reviewing previous studies and included predictors used in several predictive models and risk factors listed in the literature review, with the addition of some commonly used laboratory test^6,7,16,17,28,29^. Data were pre-collected in the clinical information system to identify accessible predictors, minimizing the occurrence of missing values in the formal data collection phase. Several laboratory test indices such as blood lactate and arterial blood pH, were not included in previous studies. Given that these indicators are common in clinical settings, this study sought to explore their potential predictive value. To make it easier for healthcare workers to use the model in the future, we defined all laboratory tests as dichotomous variables based on the range of medical normal values.

The study included 28 candidate predictors, categorized as follows: 3 demographic, 8 disease-related, 2 therapy-related, 4 medication, and 11 laboratory indicators. According to previous studies, all the predictors will be obtained in clinical practice within 48 h of admission to the ICU, with the expectation that the model will be utilized to make predictions at this time point to promote early recovery and prevent functional impairment^6^. For laboratory test indicators that were measured multiple times within 48 h of admission to the ICU, the results of the first measurement were used for statistics and analyses. The study included the Acute Physiology and Chronic Health Evaluation II (APACHE II) score, which is widely used to predict in-hospital mortality^30–32^.

### Outcome

The outcome was the BI score on the seventh day after leaving the ICU, with a BI of ≤ 60 indicating functional impairment^33^. Based on previous studies, patients with BI ≤ 60 have difficulty performing basic ADL and are dependent on the care of others, so we chose BI ≤ 60 to define functional impairment^34,35^. Nurses assessed the BI on the seventh day after leaving the ICU, recording the results in the patient's nursing record. The BI was recorded as 0 if the patient died during the ICU stay or within 7 days of leaving the ICU. If the patient was discharged within 7 days of leaving the ICU, the patient was followed up via telephone.

### Development dataset and temporal validation dataset

Participants were categorized into two cohorts: those admitted to the ICU from August 1, 2022, to June 30, 2023 (development dataset), and those from July 1, 2023, to August 20, 2023 (temporal validation dataset). The development dataset was utilized for model fitting and hyperparameter tuning using tenfold cross-validation and grid search. The temporal validation dataset was employed to assess the predictive performance and generalizability of the models. Temporal validation is considered as an in-between validation of internal and external validation, as the highest standard of external validation requires validation of the derived model in patients from different temporal periods, from a different geographic area, and by different investigators^36^. Due to the challenges in obtaining robust external validation data, we can only use temporal validation to assess the predictive performance of the model in the same population. The broader applicability of predictive models needs to be validated in different populations. Missing values were present for some candidate predictors in both the development and temporal validation datasets. We employed multiple imputations using the logistic regression method in the "mice" package (R 4.2.2) to address missing values in both the development and temporal validation datasets. The proportion of missing data for each predictor is detailed in Supplementary file 1: S1.

### Statistical analysis

Continuous variables were reported as means ± standard deviations for normally distributed data and medians (interquartile ranges) for non-normally distributed data. Categorical variables were summarized as frequencies and percentages. Baseline characteristics between the functional impairment and non-functional impairment groups were compared using the Student t-test for continuous variables with a normal distribution. The Mann–Whitney U test for those without Categorical variable differences was assessed using the chi-square test or Fisher's exact test, as appropriate. A two-tailed p-value ≤ 0.05 was considered statistically significant. Confidence intervals for the AUCs of the four models were calculated using bootstrapping and statistical differences between the AUCs were compared.

### Feature selection

Feature selection was performed using the least absolute shrinkage and selection operator (LASSO) regression. To make the model more concise, we chose the subset of features selected when the error parameter was lambda.1 se (the lambda value is equal to the minimum value of the model error plus one standard error) to construct the ML model. Lambda values were determined through tenfold cross-validation.

### Model training and tuning in the internal validation dataset

We chose four models that perform well in classification tasks: (1) logistic regression, (2) RF, (3) XGBoost, and (4) SVM. For each of these four models, we used the following program packages in R 4.2.2: (1) "glm", (2) "randomForest", (3) "xgboost " and (4) "e1071".

The development dataset, containing the selected features, was used to train the four models. Hyperparameters for RF, SVM, and XGBoost were tuned using a tenfold cross-validation and grid search approach. The final models were constructed with the optimized hyperparameters. The grid search information, hyperparameter tuning results and code used for the ML model are available in Supplementary file 1: S2.

### Measuring performance in the temporal validation dataset

The models' generalization performance was assessed using the temporal validation dataset. Model performance was evaluated based on the area under the curve (AUC), accuracy, sensitivity, specificity, positive predictive value (PPV), negative predictive value (NPV), and calibration curves. To demonstrate the best classification effect, we report performance metrics such as accuracy, sensitivity, and specificity based on the optimal threshold of the Receive Operation characteristic Curve (ROC) for each model.

### Explainability

We used the DALEX package to improve the interpretability of the final best model^25^. The DALEX package measures the importance of a feature by comparing the loss in the AUC after randomly permuting the feature compared to the original AUC, with a greater loss representing higher feature importance. DALEX package-generated visualizations facilitated the interpretation of the model, enabling the evaluation of variable importance, their relationships with clinical outcomes, and their contributions to the predicted results.

### Ethical approval

This investigation was conducted in accordance with the ethical standards of the institutional and national research committee, adhering to the 1964 Helsinki Declaration and its later amendments. The study received approval from the Ethical Review Board of the First Affiliated Hospital of Shantou University Medical College (Approval number: B-2023-092; 19 June 2023).

### Consent to participate

The ethical committee of the First Affiliated Hospital of Shantou University Medical College waived the requirement for informed consent because the investigators used hospitalization serial numbers instead of patient names when collecting data.

## Results

### Patient characteristics

**Figure 1:**
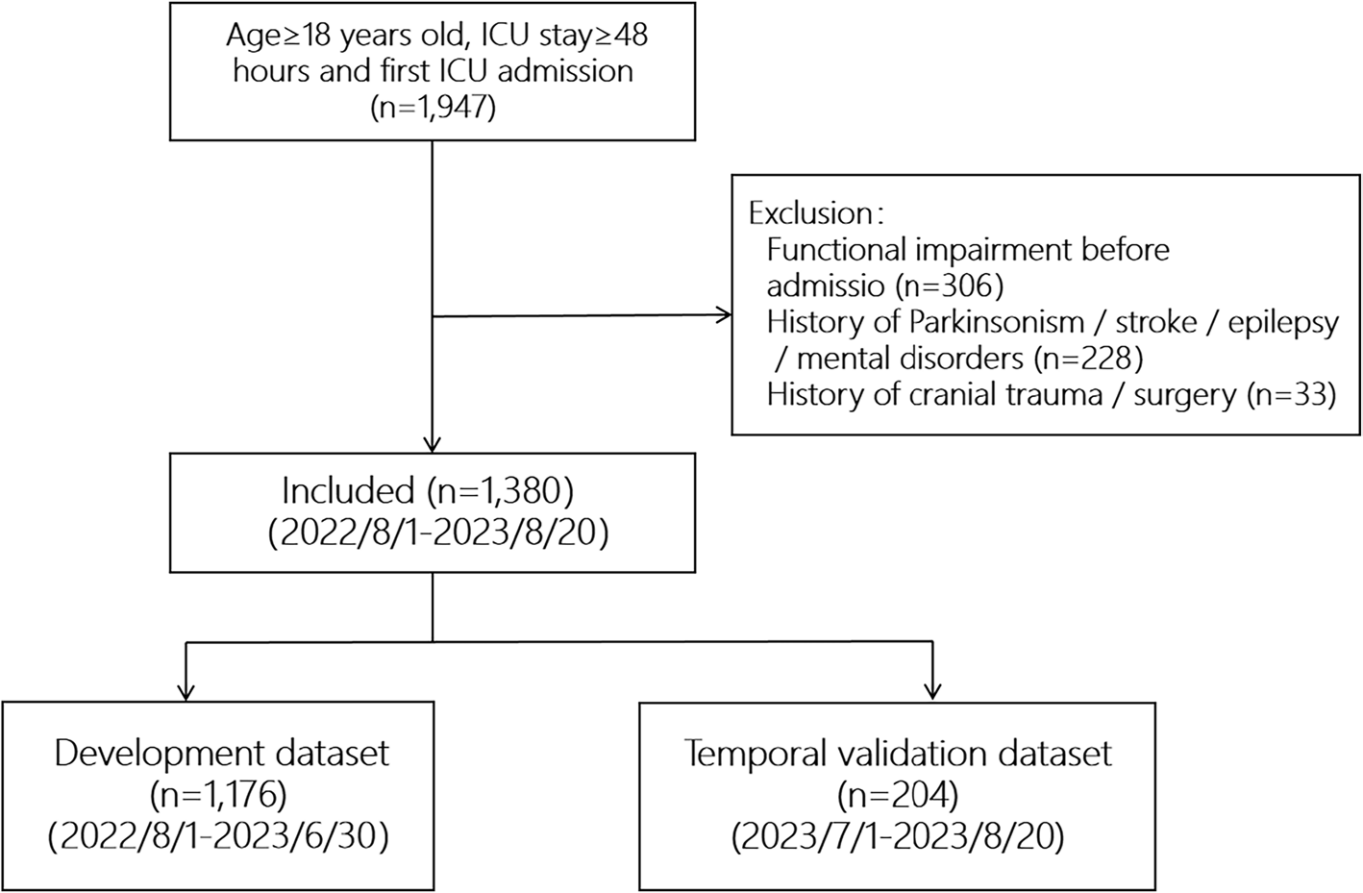
Flowchart of patient selection. Abbreviations: ICU, intensive care unit.

This study retrospectively analyzed data from 1947 patients. Excluding 567 patients (as shown in Fig. 1), the analysis included 1380 patients. The development dataset comprised 1176 patients, while the temporal validation dataset included 204 patients. Of the 1176 patients in the development dataset, 581 (49.4%) experienced new-onset functional impairment, and among the 204 in the temporal validation dataset, 103 (50.5%) had new-onset impairments. Patients were categorized into two groups based on their BI score on the seventh day after leaving the ICU: the functional impairment group (684/1380, 49.6%) and the non-impairment group (694/1380, 50.4%). Demographic and clinical characteristics for both functional impairment and non-impairment groups are presented in Table 1.

**Table 1:** Demographic and clinical characteristics between functional impairment and non-functional impairment group.

Predictors	Functional impairment (n = 684)	No functional impairment (n = 696)	*P*
Demographics			
Gender, n (%)			
Man	417 (61.0)	491 (70.5)	< 0.001
Woman	267 (39.0)	205 (29.5)	
Age	62.0 (50.0–70.0)	59.0 (47.0–68.0)	0.001
CCI	3.0 (2.0–5.0)	3.0 (1.0–4.0)	< 0.001
Disease factors			
APACHE II	18.0 (12.0–22.0)	10.0 (7.0–13.0)	< 0.001
Fracture, n (%)	118 (17.3)	11 (1.6)	< 0.001
Sepsis, n (%)	48 (7.0)	31 (4.5)	0.04
Cancer, n (%)	56 (8.2)	23 (3.3)	< 0.001
Cerebrovascular disease, n (%)	237 (34.6)	9 (1.3)	< 0.001
Head injury, n (%)	71 (10.4)	2 (0.3)	< 0.001
Operation, n (%)	428 (62.6)	365 (52.4)	< 0.001
Delirium, n (%)	104 (15.2)	25 (3.6)	< 0.001
Therapies factors			
Mechanical ventilation, n (%)	410 (59.9)	101 (14.5)	< 0.001
CRRT, n (%)	57 (8.3)	39 (5.6)	0.046
Medication factors			
Benzodiazepines, n (%)	276 (40.4)	77 (11.1)	< 0.001
Opioids, n (%)	208 (30.4)	92 (13.2)	< 0.001
Dexmedetomidine, n (%)	225 (32.9)	97 (13.9)	< 0.001
Propofol, n (%)	163 (23.8)	46 (6.6)	< 0.001
Laboratory indicators			
Blood pH > 7.45 or pH < 7.35, n (%)	465 (68.0)	318 (45.7)	< 0.001
Blood lactate > 2.0 mmol/L, n (%)	371 (54.2)	207 (29.7)	< 0.001
CRP > 8.00 mg/L, n (%)	610 (89.2)	503 (72.3)	< 0.001
Albumin < 40.00 g/L, n (%)	609 (89.0)	549 (78.9)	< 0.001
BUN > 7.60 mmol/L, n (%)	268 (39.2)	250 (35.9)	0.211
Creatinine > 133 μmol/L, n (%)	164 (24.0)	143 (20.5)	0.126
ALT > 40.00U/L, n (%)	163 (23.8)	209 (30.0)	0.009
AST > 40.00U/L, n (%)	276 (40.4)	353 (50.7)	< 0.001
Leucocyte < 3.50 × 10^9^/L or > 9.5 × 10^9^/L, n (%)	490 (71.6)	428 (61.5)	< 0.001
Erythrocyte < 3.80 × 1012/L, n (%)	511 (74.7)	399 (57.3)	< 0.001
Hemoglobin < 115 g/L, n (%)	536 (78.4)	417 (59.9)	< 0.001

*CCI* Charlson Comorbidity Index, *APACHE II* Acute Physiology and Chronic Health Evaluation II score, *CRRT* continuous renal replacement therapy, *CRP* C-reactive protein, *BUN* blood urea nitrogen, *ALT* alanine aminotransferase, *AST* aspartate transaminase.

### Feature selection

**Figure 2:**
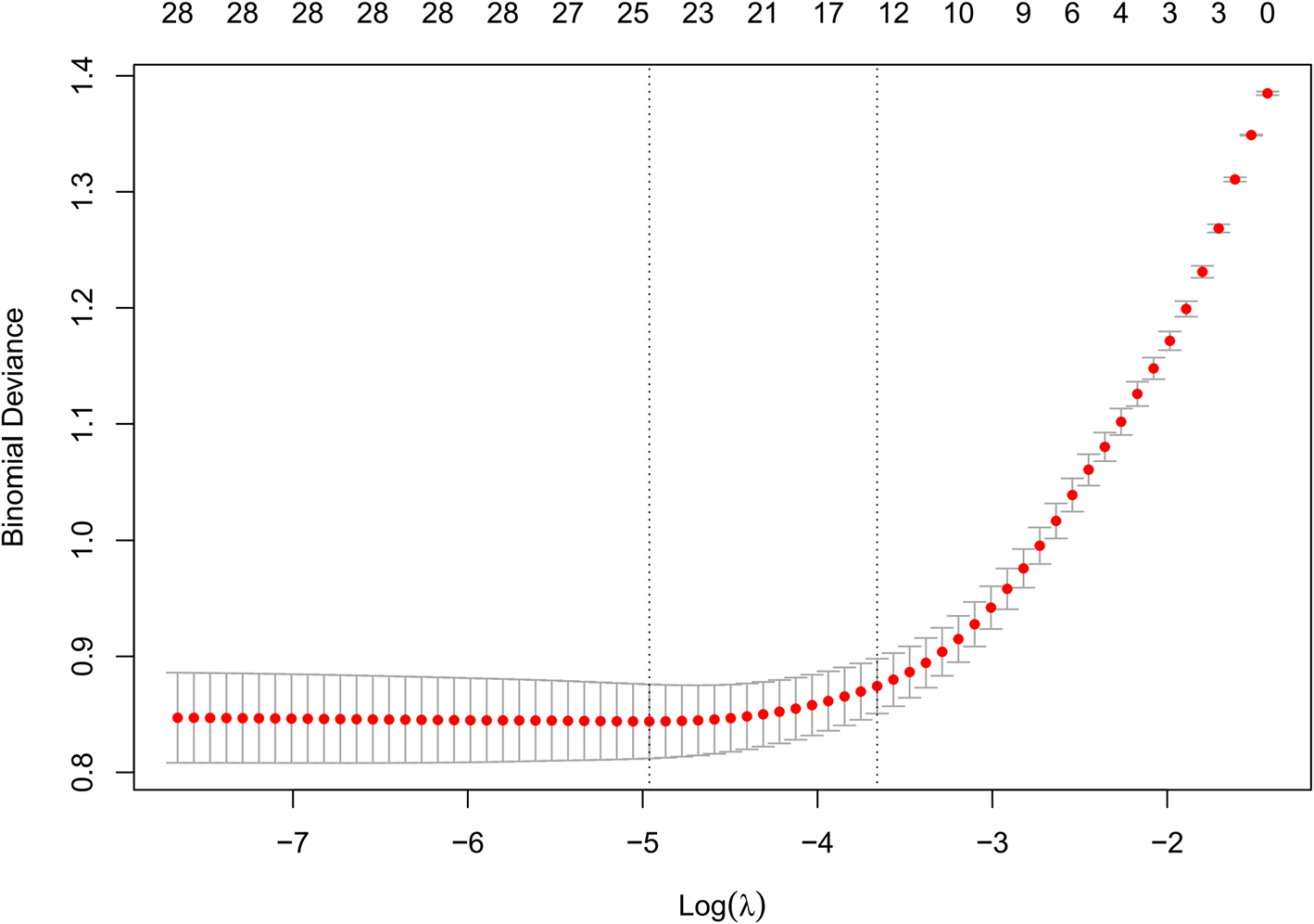
Selection of predictors using LASSO regression.

Our study collected data on 28 potential predictors within 48 h of ICU admission. We employed LASSO regression for feature selection, identifying 13 significant variables (Fig. 2). The variables that were used to construct the model include Charlson Comorbidity Index (CCI), APACHE II, Fracture, Cerebrovascular disease, Head injury, Delirium, MV, Benzodiazepines, Dexmedetomidine, Blood pH, Blood lactate, C-reactive protein (CRP), Hemoglobin.

### Performance of the models in the temporal validation dataset

**Figure 3:**
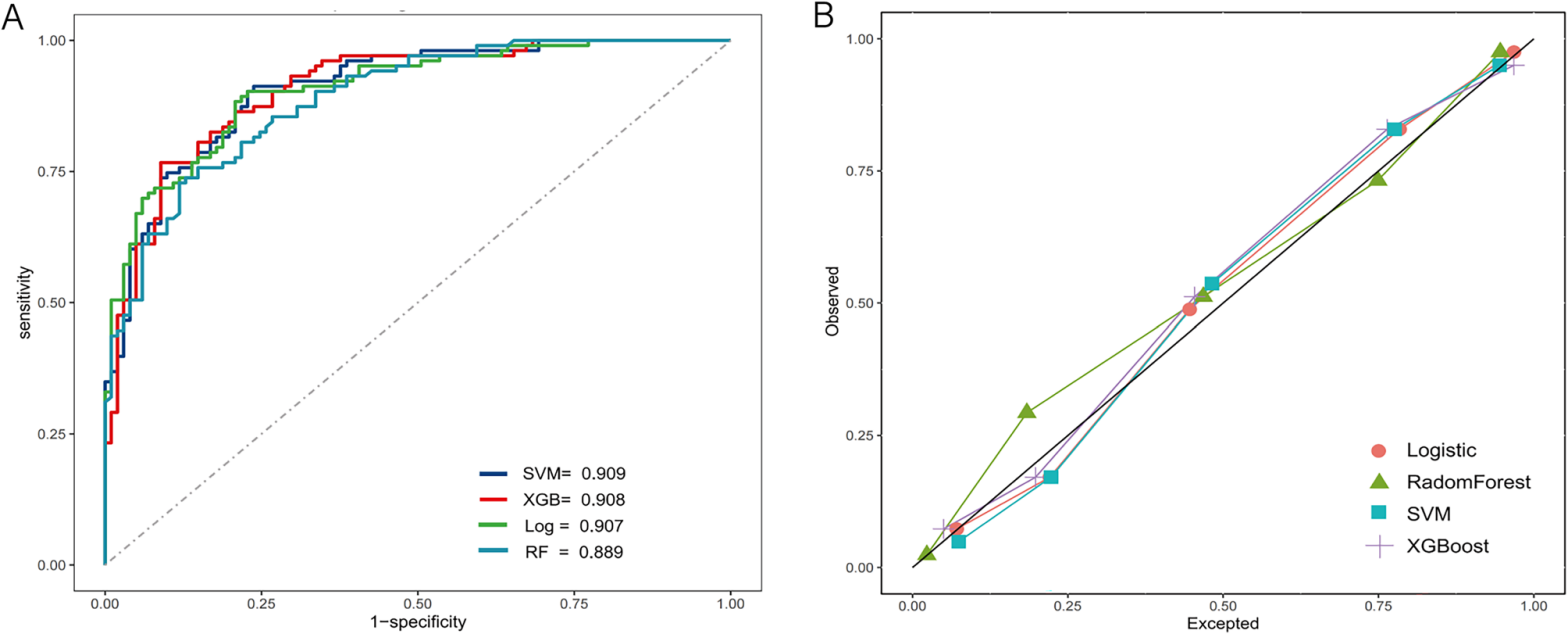
Performance of the four models in the temporal validation dataset (**A** Receiver operating characteristic curves (ROC), **B** calibration plot).

Table 2 and Fig. 3 display the performance of the four models on the temporal validation dataset. The four models demonstrated good discriminative ability with AUC values above 0.88, with the SVM model achieving the highest AUC of 0.909. Based on the best thresholds for each model, the SVM and logistic regression have high sensitivity (0.88–0.90) and moderate specificity (0.77–0.79), while the XGBoost and RF have high specificity (0.88–0.91) and moderate sensitivity (0.72–0.76). Sensitivity can be used to measure the ability of a predictive model to recognize positive events (new onset functional impairment). A comparison of the AUCs of the four models by bootstrapping revealed no significant differences in AUCs between the models. The calibration curves for all the models were distributed around the calibration line and the calibration was satisfactory (Fig. 3B).

**Table 2:** Performance of the four models in the temporal validation dataset.

Model	AUC (95%CIs)	Threshold	Accuracy	Sensitivity	Specificity	PPV	NPV
SVM	0.909 (0.869–0.947)	0.621	0.838	0.902	0.772	0.802	0.886
XGBoost	0.908 (0.868–0.947)	0.564	0.838	0.767	0.911	0.898	0.793
Logistic	0.907 (0.868–0.947)	0.371	0.838	0.883	0.792	0.813	0.869
RF	0.889 (0.846–0.931)	0.568	0.804	0.728	0.881	0.862	0.761

*SVM* support vector machine, *XGBoost* extreme gradient boosting, *RF* random forest, *AUC* area under the curve, *PPV* positive predictive value, *NPV* negative predictive value.

### Explainability

**Figure 4:**
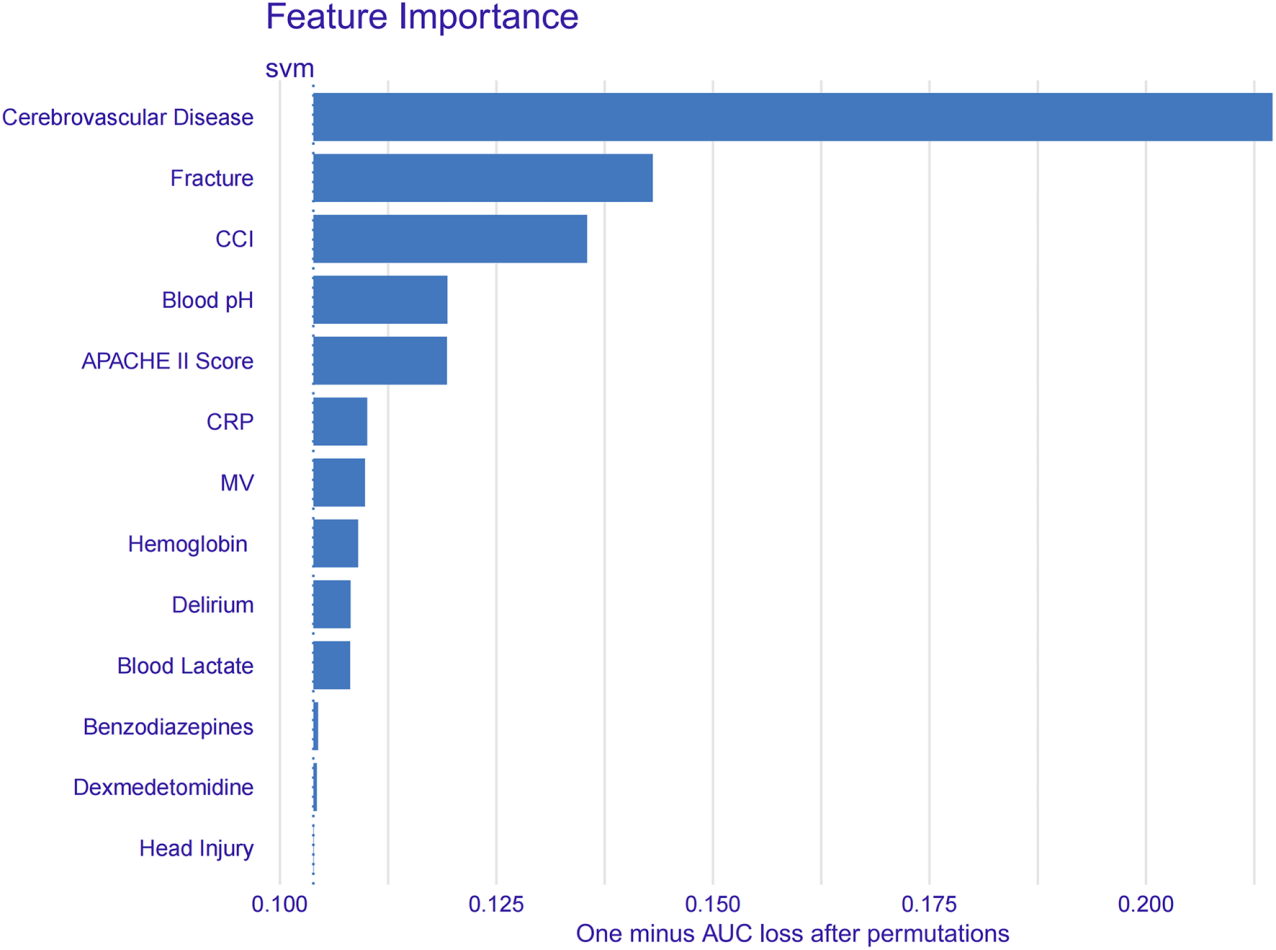
Feature importance derived from support vector machine model. This figure is the result of the DALEX package. The X-axis represents the loss in the area under the curve (AUC) calculated after randomly permuting the feature compared to the original AUC. The greater this loss, the higher the model’s importance of this feature. CCI, Charlson Comorbidity Index; APACHE II score, Acute Physiology and Chronic Health Evaluation II score; CRP C-reactive protein; MV, mechanical ventilation.

We calculated the feature importance using the DALEX package and Fig. 4 represents the importance of each feature in the SVM model. The figure shows that cerebrovascular disease is the most important predictor of new-onset functional impairment in critically ill patients. In addition, the importance of predictors such as fracture, CCI, etc. can be seen in Fig. 4.

**Figure 5:**
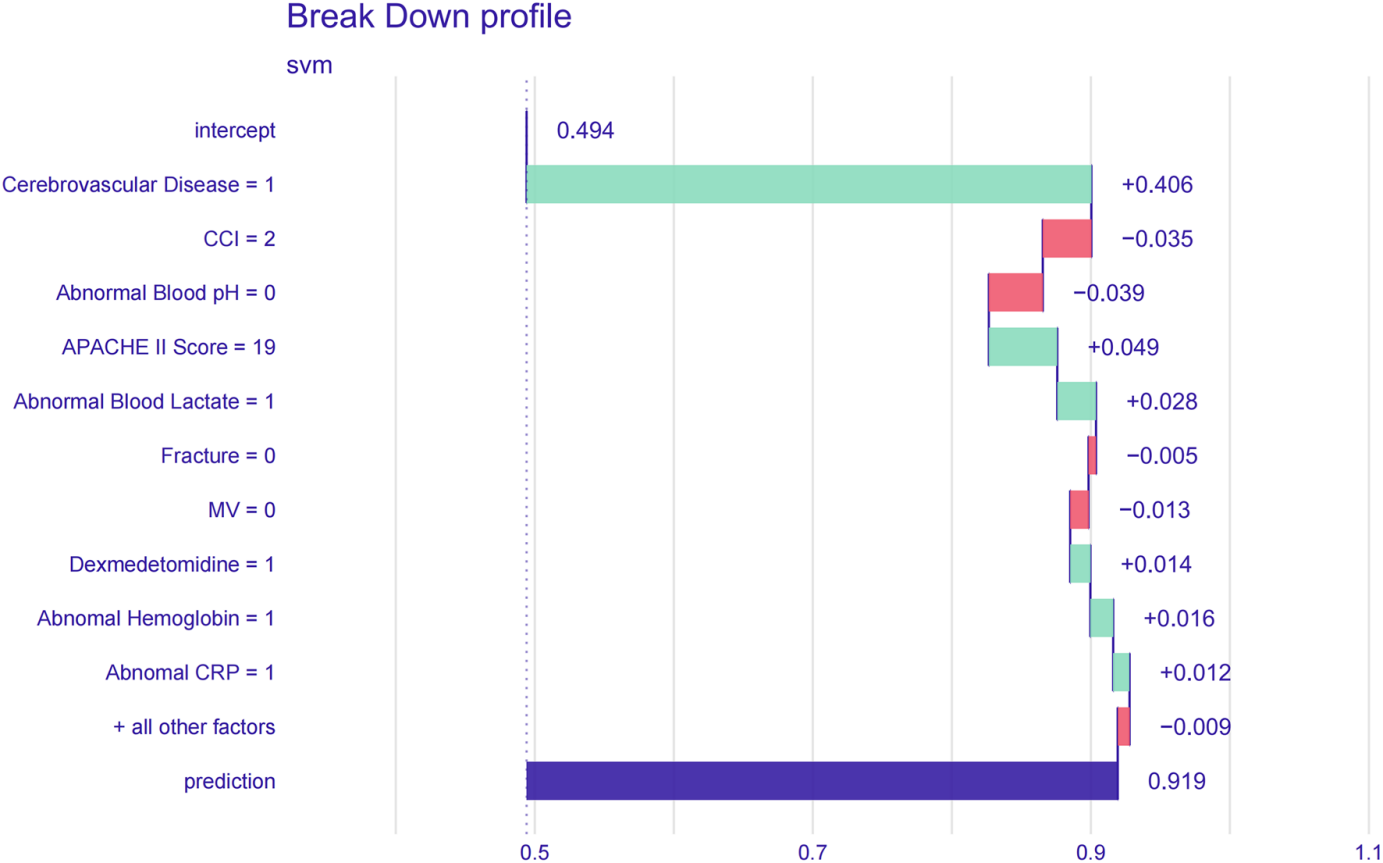
This figure was made with the DALEX package to explain support vector machine model predictions. The numbers on the X-axis indicate the predicted probability of new-onset functional impairment obtained from the model's estimation based on a patient's features. CCI, Charlson Comorbidity Index; APACHE II score, Acute Physiology and Chronic Health Evaluation II score; MV, mechanical ventilation; CRP C-reactive protein.

In addition to calculating feature importance, we selected one patient's data for individual interpretation (Fig. 5). Figure 5 indicates that cerebrovascular disease, an APACHE II score of 19, abnormal lactate and hemoglobin levels, CRP, and dexmedetomidine use raised the patient's risk for new-onset functional impairment, while a CCI of 2, normal blood pH, no fracture diagnosis, and no MV use mitigated this risk. This patient's calculated risk for new-onset functional impairment was 0.919.

## Discussion

### Principal findings

In this study, we developed four predictive models based on 13 variables collected within 48 h of ICU admission to predict whether a critically ill patient had a new onset of functional impairment on day 7 after leaving the ICU. The SVM model exhibited superior performance. Feature importance analysis revealed the following variables as the most influential in the SVM model, listed in order of importance: cerebrovascular disease, fracture, CCI, blood pH, APACHE II, CRP, MV, hemoglobin, delirium, lactate, benzodiazepines, dexmedetomidine, and head injury. Ultimately, the ML model's interpretable algorithm elucidates the prediction process for individual cases.

### Related works

Before our study, four analogous studies were published. A study by Japanese scholars developed prediction models for new-onset functional impairment at discharge of ICU inpatients with a large sample (n = 19,846) and obtained good predictive results (AUC > 0.86)^6^. The research design and modeling process of this study are relatively sound, and our study design draws in part on that study. Another US multicenter study predicts functional outcomes in elderly patients 6 months after ICU discharge^18^. A 2017 prospective study in the US explored predictors of failure to return to baseline functional status in critically ill patients at 6 months after hospital discharge^17^. A 2014 Swedish prospective study aimed to predict discharge functional status among middle-aged patients^7^. Given the small sample sizes, shortage of external validation, and incomplete predictors in the last three studies, their models may not be clinically applicable.

Compared to previous studies, the main advantage of our study is the construction of interpretable models. The ML model constructed in the first study also has excellent predictive performance^6^, but relying only on feature importance may not allow users to fully understand the decision basis of the model, which limits its application in clinical settings. Our interpretable ML models enhance users' understanding of the decision-making process, increasing reliability and transparency. In addition to reporting the importance of the features of the best models, our models provide parameters for the contribution of predictors to individual predictions and positive and negative tendencies, which help caregivers to develop more flexible care plans based on the patient's specific situation. Additionally, our model's strong classification performance (AUC > 0.88) enables effective identification of patients at high risk of functional impairment, prioritizing healthcare resources for early intervention and optimizing their allocation. Concurrently, this approach enhances the quality of life for critically ill patients. Strengthening monitoring and early intervention for high-risk patients helps reduce the incidence of functional impairment.

Compared with the above studies, our study has some similarities. First, we collected clinical data within 48 h of ICU admission for critically ill patients. This approach aimed to facilitate early prediction and prompt intervention. It has been suggested that implementing early mobilization and neuromuscular electrical stimulation treatments within 2 days of a patient's admission to the ICU reduces muscle loss and improves muscle strength and physical mobility^37–39^. Second, our final model included some predictors that aligned with previous studies, such as cerebrovascular disease, fracture, MV, APACHE II, CCI, and CRP, which were identified as candidate predictors by referring to related studies and literature review before the study started. Lastly, the ML models, model performance, and validation methods in this study were similar to those in previous studies^6^. Given the binary outcome of this study, we selected ML models (RF, SVM, XGBoost) known for their strong performance in previous research for enhanced predictive accuracy^26^. This consistency in model performance reaffirms the predictive value of the selected predictors. Owing to data limitations, we also utilized a temporal validation dataset for external performance assessment of the models.

However, our study differs from previous ones in several aspects. First of all, the time point for predicting functional impairment in this study was day 7 after the patient left the ICU, whereas in similar studies patient discharge was chosen as the time point for the outcome measure^6^. We chose this time point for three reasons: firstly, evidence suggests a correlation between the activity level of critically ill patients during their ICU stay and the activity level of patients within 7 days after they leave the ICU^40^, and we expect to guide some nursing decisions during ICU stay by predicting the functional status of patients on the 7th day after they leave the ICU. Secondly, according to our fieldwork, the time it takes for critically ill patients to leave the ICU and be discharged is usually greater than 7 days. The longer this period, the more external factors not related to the ICU may affect the patient's functional status, potentially reducing prediction accuracy. Thirdly, because most patients are still receiving care in the hospital on day 7 after leaving the ICU, nurses can assess the patient's functional status in the field to ensure the veracity of the outcome indicators. Furthermore, our model's performance (AUC > 0.88) surpassed that of previous studies (0.71–0.86), likely due to the selection of more predictively potent variables and a more effective feature selection approach. Our study included predictors from previous models, along with commonly used ICU lab tests and sedative medications like blood pH, lactate, benzodiazepines, and dexmedetomidine. We employed LASSO regression for feature selection. LASSO regression simplifies the model, enhances statistical efficiency, and mitigates overfitting^41^. Lastly, In our study, cerebrovascular disease was the most important predictor in the model, however it was not in the study by Ohbe^6^. In our study, patients admitted to the neurosurgical intensive care unit (NSICU) were a special and important group, most of these patients were diagnosed with cerebrovascular disease. In this study, the prevalence of functional impairment in this population reached 89.9% (170/189). A total of 189 NSICU patients were included in this study, representing 13.7% (189/1380) of all patients. The inclusion of these NSICU patients may have contributed to cerebrovascular disease being the most important predictor.

In our study, cerebrovascular disease, fracture, CCI, APACHE II, MV, delirium, and CRP were significant in predicting functional impairment in critically ill patients, which is consistent with previous findings. It has been reported that patients with cerebrovascular disease have a higher prevalence of functional impairment and a longer period of impact, and such patients require more functional rehabilitation^42^. Fracture is one of the main causes of short-term motility impairment and predisposes to new-onset functional impairment in critically ill patients. Higher CCI and APACHE II scores are associated with an increased risk of functional impairment, highlighting the significance of disease severity in influencing functional outcomes, consistent with systematic review findings^15^. Both MV and delirium have been shown in previous studies to be major predictors of functional impairment, with mechanisms of action associated with higher sedative medication use and altered cognitive function^43,44^. Elevated CRP indicates the presence of an infection in the patient, and studies have pointed out that severe infections can develop into a systemic inflammatory response syndrome leading to muscle protein hydrolysis, which is a potential mechanism for ICU-acquired weakness, which in turn induces functional impairment^45,46^. In addition, several new predictors were used in this study, including blood pH, blood lactate, hemoglobin, benzodiazepines, dexmedetomidine, and head injury. Abnormal blood pH, lactate, and hemoglobin levels typically signal internal environmental disruptions, hypoxia, and possible blood loss or anemia in patients. However, the mechanisms by which these several predictors promote the onset of functional impairment are currently unclear and may overlap somewhat with the predictive value of disease severity. Benzodiazepines and dexmedetomidine are commonly used for sedation in the ICU, and studies have linked the use of sedation in the ICU to post-traumatic stress syndrome, delirium, anxiety, depression, and cognitive impairment in patients^47,48^. Long-term sedation use can reduce active movement, potentially causing muscle atrophy and subsequent functional impairment^49^. Head injury ranked lowest in feature importance in this study. Severe head injuries often co-occur with cerebrovascular disease and fractures, contributing to long-term functional impairment^50^. However, moderate head injuries may have little effect on the functional status of patients. Future studies may need to conduct categorical analyses of head injury severity for more precise predictive value assessment. Our model did not incorporate age and sepsis, which are commonly used predictors in other studies^6^. A cohort study of critically ill patients aged 60–79 years and ≥ 80 years noted no difference in the degree of functional decline between the younger (60–79 years) and older (≥ 80 years) groups within 6 months of discharge^51^. This suggests that functional outcomes in older patients may vary individually. Sepsis, on the other hand, was not selected as a predictor, possibly related to the small number of septic patients in this study (5.7%, 79/1380).

## Limitations and enhancements

This study has several limitations. Firstly, the model validation method used in this study was temporal validation, which prevented us from evaluating the predictive performance of the model in different populations^52^. Secondly, although our sample size met the sample recommendation suggested by PROBAST^53^, with an event per variable (EPV) > 20 (581/28), which could avoid overfitting to a certain extent, however, it is easy to notice that the number of cases of some variables in this dataset is relatively small, such as sepsis and head injury. This may affect the results of feature selection, which in turn affects the generalization ability and predictive performance of the model. Finally, our classification criteria for variables like cerebrovascular disease and head injury are broad. The broad classification criteria may lead to poor model performance in the prediction of some specific individuals, such as patients with milder head injuries may overestimate the risk of functional impairment.

## Further research

In future research, we plan to continue to expand the sample size and further refine the variable classification criteria to improve the model performance. After the model has undergone rigorous external validation, we plan to embed the model into the hospital's electronic information system for automatic prediction. Upon completion of these tasks, our model will be defined as predictive AI^53^. It can automatically extract clinical data, automatically calculate the probability of new functional impairment in critically ill patients through machine learning model and provide the degree and direction of influence of each variable on the outcome event. These features will powerfully help clinicians and nurses to identify patients in need of intervention at an early stage, develop individualized interventions, and efficiently improve the functional outcomes of critically ill patients.

## Conclusion

ML models are reliable tools for predicting new-onset functional impairments in critically ill patients. Notably, the SVM model emerged as the most effective, enabling early identification of patients at high risk and facilitating the implementation of timely interventions to improve ADL.

### Supplementary Information

Supplementary Information 1.

Supplementary Information 2.

Supplementary Information 3.

## Data Availability

The Supplementary file 1 provides the missing data proportions and hyperparameter tuning methods (including codes) for the predictors. Supplementary file 2 and Supplementary file 3 provide the development dataset and the temporal validation dataset. All Supplementary file have been uploaded in the Supplementary Material.
