# Supplementary material for: Development and validation of early prediction models for new-onset functional impairment in patients after being transferred from the ICU
Source: Sci Rep. 2024 May 24;14:11902. doi: 10.1038/s41598-024-62447-8 (PMC11126674; doi:10.1038/s41598-024-62447-8)
Supplement: Supplementary file 1 — Supplementary Information 1. [file 41598_2024_62447_MOESM1_ESM.docx]

**S1**  Proportion of missing data for predictors

| **Predictors** | **Missing** |
| --- | --- |
| Gender, n (%) |  |
| Man | 0 |
| Woman | 0 |
| Age, n (%) | 0 |
| CCI, n (%) | 0 |
| APACHE Ⅱ, n (%) | 0 |
| Fracture, n (%) | 0 |
| Sepsis, n (%) | 0 |
| Cancer, n (%) | 0 |
| Cerebrovascular disease, n (%) | 0 |
| Head injury, n (%) | 0 |
| Operation, n (%) | 0 |
| Delirium, n (%) | 0 |
| Mechanical ventilation, n (%) | 0 |
| CRRT, n (%) | 0 |
| Benzodiazepines, n (%) | 0 |
| Opioids, n (%) | 0 |
| Dexmedetomidine, n (%) | 0 |
| Propofol, n (%) | 0 |
| Blood pH, n (%) | 121 (8.77%) |
| Blood lactate, n (%) | 121 (8.77%) |
| CRP, n (%) | 114 (8.26%) |
| Albumin, n (%) | 19 (1.38%) |
| BUN, n (%) | 9 (0.65%) |
| Creatinine, n (%) | 6 (0.43%) |
| ALT, n (%) | 21 (1.52%) |
| AST, n (%) | 20 (1.45%) |
| Leucocyte, n (%) | 0 |
| Erythrocyte, n (%) | 0 |
| Hemoglobin, n (%) | 0 |

*CCI* Charlson Comorbidity Index, *APACHE Ⅱ* Acute Physiology and Chronic Health Evaluation II score, *CRRT* continuous renal replacement therapy, *CRP* C-reactive protein, *BUN* blood urea nitrogen, *ALT* alanine aminotransferase, *AST* aspartate transaminase

**S2**

1.RF’s grid search and hyperparameters result :

mtry:from 1 to 51, by 5 ; result: mtry=6

ntree:100,500,1000 ; result: ntree=1000

**Code:**

ctrl <- trainControl(method = "cv", number = 10)

grid <- expand.grid(mtry = seq(from = 1, to = 51, by = 5))

rf_model <- train(x = X_train, y = y_train,

method = "rf",

trControl = ctrl,

tuneGrid = grid)

grid <- expand.grid(mtry = c(6))

modellist <- list()

set.seed(1234)

for (ntree in c(100,500,1000)) {

fit <- train(x = X_train, y = y_train, method="rf",

metric="Accuracy", tuneGrid=grid,

trControl=ctrl, ntree=ntree)

key <- toString(ntree)

modellist[[key]] <- fit

}

results <- resamples(modellist)

summary(results)

final_model <- randomForest(x = X_train, y = y_train,mtry = 6,ntree = 1000)

print(final_model)

1. XGBoost’s grid search and hyperparameters result :

Adjustment of hyperparameters using tenfold cross-validation and automatic grid search.Refer to the following code for details.

nrounds =50, max_depth = 3, eta=0.3,

gamma=0,colsample_bytree=0.6,

min_child_weight=1, subsample=1

**Code:**

set.seed(1234)

fitControl = trainControl(method = "cv", number = 10, search = "grid" )

caret_xgb = train(ADL~CCI+APACHESCORE+FRACTURE+CEREBROVASCULAR_DISEASE

+DELIRIUM+MECHANICAL_VENTILATION+BENZODIAZEPINES

+DEXMEDETOMIDINE+PH_ABNORMAL+LACTIC+HEAD_INJURY

+HEMOGLOBIN_ABNORMAL+CRP,

data = result,

method="xgbTree",

trControl=fitControl,

nthread = 8

)

caret_xgb$bestTune

xgb_mod <- xgb.train(data = train_data, objective = "binary:logistic",

nrounds =50,max_depth = 3,eta=0.3,

gamma=0,colsample_bytree=0.6,

min_child_weight=1, subsample=1)

1. SVM’s grid search and hyperparameters result :

kernel: "linear", "polynomial", "radial", "sigmoid"; result:kernel = "linear"

(The optimal kernel function is selected by comparing the accuracy of models constructed with different kernel functions.)

cost :0.001, 0.01, 0.1, 1, 5, 10; result: cost=0.1

**Code:**

linear.tune <- tune.svm(ADL~CCI+APACHESCORE+FRACTURE+CEREBROVASCULAR_DISEASE

+DELIRIUM+MECHANICAL_VENTILATION+BENZODIAZEPINES

+DEXMEDETOMIDINE+PH_ABNORMAL+LACTIC+HEAD_INJURY

+HEMOGLOBIN_ABNORMAL+CRP, data = result,

kernel = "linear",

cost = c(0.001, 0.01, 0.1, 1, 5, 10),probability=TRUE)

summary(linear.tune)

best.linear <- linear.tune$best.model #cost=0.1;best performance: 0.1870636

linear.test <- predict(best.linear, newdata = result1)

linear.table<-table(linear.test, result1$ADL)

accuacy_rate <- sum(diag(linear.table)) / sum(linear.table)

print(paste0("Accuracy rate: ", accuacy_rate)) #0.813725490196078"

poly.tune <- tune.svm(ADL~CCI+APACHESCORE+FRACTURE+CEREBROVASCULAR_DISEASE

+DELIRIUM+MECHANICAL_VENTILATION+BENZODIAZEPINES

+DEXMEDETOMIDINE+PH_ABNORMAL+LACTIC+HEAD_INJURY

+HEMOGLOBIN_ABNORMAL+CRP,data = result,

kernel = "polynomial",

degree = c(3, 4, 5),

coef0 = c(0.1, 0.5, 1, 2, 3, 4))

summary(poly.tune)

best.poly <- poly.tune$best.model #degree=3,coef0=0.5;best performance: 0.1878966

poly.test <- predict(best.poly, newdata = result1)

poly.table<-table(poly.test, result1$ADL)

sum(diag(poly.table)/sum(poly.table))

## 0.8039216

rbf.tune <- tune.svm(ADL~CCI+APACHESCORE+FRACTURE+CEREBROVASCULAR_DISEASE

+DELIRIUM+MECHANICAL_VENTILATION+BENZODIAZEPINES

+DEXMEDETOMIDINE+PH_ABNORMAL+LACTIC+HEAD_INJURY

+HEMOGLOBIN_ABNORMAL+CRP,data = result,

kernel = "radial",

gamma = c(0.1, 0.5, 1, 2, 3, 4))

summary(rbf.tune)

best.rbf <- rbf.tune$best.model ## gamma=0.1

rbf.test <- predict(best.rbf, newdata = result1)

rbf.table<-table(rbf.test, result1$ADL)

sum(diag(rbf.table)/sum(rbf.table))

##0.8137255

sigmoid.tune <- tune.svm(ADL~CCI+APACHESCORE+FRACTURE+CEREBROVASCULAR_DISEASE

+DELIRIUM+MECHANICAL_VENTILATION+BENZODIAZEPINES

+DEXMEDETOMIDINE+PH_ABNORMAL+LACTIC+HEAD_INJURY

+HEMOGLOBIN_ABNORMAL+CRP,data = result,

kernel = "sigmoid",

gamma = c(0.1,0.2,0.3, 0.4,0.5),

coef0 = c(0.1, 0.5, 1, 2, 3, 4),

probability=TRUE)

summary(sigmoid.tune)

best.sigmoid <- sigmoid.tune$best.model

sigmoid.test <- predict(best.sigmoid, newdata = result1,positive = "1") #gamma=0.1 coef0=2

sigmoid.table<-table(sigmoid.test, result1$ADL)

sum(diag(sigmoid.table)/sum(sigmoid.table))

## 0.7352941
